# Supplementary material for: Extended field-of-view ultrathin microendoscopes for high-resolution two-photon imaging with minimal invasiveness
Source: eLife. 2020 Oct 13;9:e58882. doi: 10.7554/eLife.58882 (PMC7685710; doi:10.7554/eLife.58882)
Supplement: Supplementary file 1. — Supplementary Table 1: Parameters for the fabrication of corrective lenses. Coefficients used in Equation (1) (see Materials and methods) for the aspherical corrective lenses used in type I-IV eFOV-microendoscopes. Supplementary Table 2: Simulated focal length in uncorrected and corrected microendoscopes. Supplementary Table 3: Spatial resolution and effective FOV of eFOV-microendoscopic probes. Values are reported as average ± sem. For statistical comparison of uncorrected (uncor.) vs. corrected (cor.) microendoscopes, Student’s t-test was used. Supplementary Table 4: Statistical comparisons of behavior state distributions as a function of pupil diameter. For the statistical comparison of Q, W, and WL state distributions in each range of pupil diameter, a two-way ANOVA with Tukey-Kramer post-hoc correction was performed. [file elife-58882-supp1.docx]

|  | **c** | **k** | **α1** | **α2** | **α3** | **α4** | **α5** | **α6** | **α7** | **α8** |
| --- | --- | --- | --- | --- | --- | --- | --- | --- | --- | --- |
| **Type I** | -2.6E-01 | -1.7E+00 | 8.6E-01 | -5.3E+01 | 6.0E+03 | -2.77E+05 | 7.3E+06 | -8.9E+07 | 2.5E+08 | 2.2E+09 |
| **Type II** | 1.0E+03 | -2.4E+03 | -1.5E+00 | 2.9E+01 | -4.6E+01 | -7.69E+03 | 2.0E+05 | 7.8E+06 | -1.7E+08 | -3.9E+08 |
| **Type III** | -5.0E-01 | 8.2E+00 | 1.5E+00 | -1.4E+02 | 3.7E+04 | -3.92E+06 | 2.2E+08 | -5.6E+09 | 3.3E+10 | 6.3E+11 |
| **Type IV** | -2.4E-01 | -1.2E+00 | 9.2E-01 | -1.3E+02 | 4.4E+04 | -4.28E+06 | 2.2E+08 | -5.6E+09 | 3.5E+10 | 4.9E+11 |

**Supplementary Table 1. Parameters for the fabrication of corrective lenses.** Coefficients used in equation (*1*) (see Materials and methods) for the aspherical corrective lenses used in type I-IV *eFOV*-microendoscopes.

| Type | Focal length in uncorrected microendoscopes (µm) | Focal length in *eFOV-* microendoscopes (µm) |
| --- | --- | --- |
| I | 190 | 188 |
| II | 194 | 201 |
| III | 195 | 172 |
| IV | 237 | 226 |

**Supplementary Table 2. Simulated focal length in uncorrected and corrected microendoscopes.**

| Type | On axis FWHM_x,y_  (µm)  n ≥ 8 | On axis FWHM_z_  (µm)  n ≥ 8 | Effective FOV radius  (µm) | Fold increase in FOV area |
| --- | --- | --- | --- | --- |
| I | uncor.: 0.91 ± 0.01  cor.: 0.94 ± 0.02  p = 0.1607 | uncor.: 9.54 ± 0.18  cor.: 8.85 ± 0.12  p = 0.0026 | uncor.: 74  cor.: 133 | 3.2 |
| II | uncor.: 0.80 ± 0.01  cor.: 0.87 ± 0,02  p = 0.0064 | uncor.: 8.91 ± 0.15  cor.: 8.87 ± 0,11  p = 0.7916 | uncor.: 51  cor.: 156 | 9.4 |
| III | uncor.: 0.88 ± 0.02  cor.: 0.89 ± 0.00  p = 0.4298 | uncor.: 7.72 ± 0.07  cor.: 7.45 ± 0.16  p = 0.1861 | uncor.: 62  cor.: 154 | 6.2 |
| IV | uncor.: 0.78 ± 0.02  cor.: 0.89 ± 0.03  p = 0.0041 | uncor.: 8.86 ± 0.24  cor.: 8.32 ± 0.29  p = 0.1583 | uncor.: 34  cor.: 69 | 4.1 |

**Supplementary Table 3. Spatial resolution and effective FOV of *eFOV-*microendoscopic probes.** Value are reported as average ± sem. For statistical comparison of uncorrected (uncor.) *vs* corrected (cor.) microendoscopes, Student’s *t*-test was used.

| Pupil  diameter  range | 0-0.1 | 0.1-0.2 | 0.2-0.3 | 0.3-0.4 | 0.4-0.5 | 0.5-0.6 | 0.6-0.7 | 0.7-0.8 | 0.8-0.9 | 0.9-1.0 |
| --- | --- | --- | --- | --- | --- | --- | --- | --- | --- | --- |
| Q vs W | p = 1 | p = 1 | p = 1 | p = 1 | p = 1 | p = 1 | p = 0.7 | p = 1 | p = 1 | p = 1 |
| Q vs WL | p = 1 | p = 0.04 | p = 7E-5 | p = 2E-6 | p = 2E-4 | p = 0.7 | p = 1 | p = 0.009 | p = 2E-6 | p = 2E-6 |
| W vs WL | p = 1 | p = 0.8 | p = 0.9 | p = 0.002 | p = 0.04 | p = 0.2 | p = 1 | p = 0.8 | p = 2E-6 | p = 2E-6 |

**Supplementary Table 4. Statistical comparisons of behavior state distributions as a function of pupil diameter.** For the statistical comparison of Q, W, and WL state distributions in each range of pupil diameter, a two-way ANOVA with Tukey-Kramer *post hoc* correction was performed.
